# Supplementary figures and images for: Allometric equation for Raphia laurentii De Wild, the commonest palm in the central Congo peatlands
Source: PLoS One. 2023 Apr 14;18(4):e0273591. doi: 10.1371/journal.pone.0273591 (PMC10104305; doi:10.1371/journal.pone.0273591)

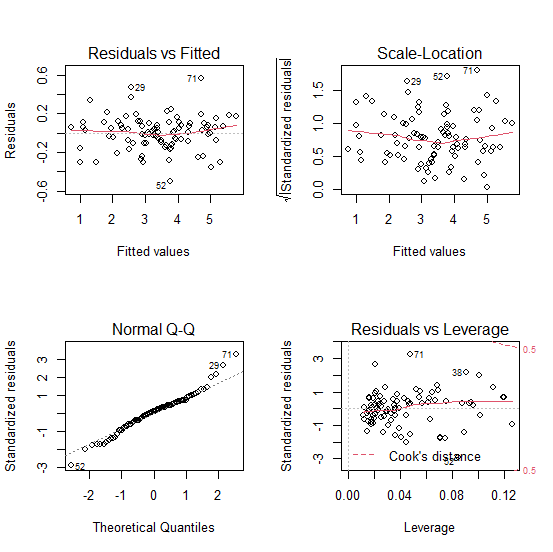


S1 Fig. Residual graphs of the model 11

Supplement: S1 Fig — (DOCX) [file pone.0273591.s001.docx]

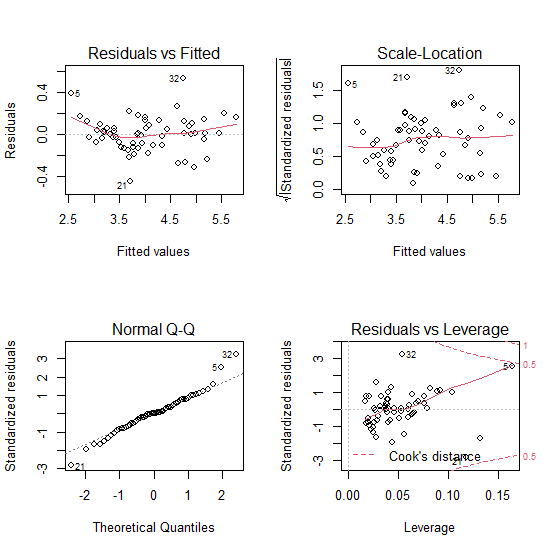


S2 Fig. Residual graphs of the model 18

Supplement: S2 Fig — (DOCX) [file pone.0273591.s002.docx]
