# Supplementary material for: Allometric equation for Raphia laurentii De Wild, the commonest palm in the central Congo peatlands
Source: PLoS One. 2023 Apr 14;18(4):e0273591. doi: 10.1371/journal.pone.0273591 (PMC10104305; doi:10.1371/journal.pone.0273591)
Supplement: S1 Table — (DOCX) [file pone.0273591.s003.docx]

S1 Table. Tissue density values of the different compartments of an individual *Raphia laurentii*

| **Palm compartment** | **Mean palm diameter range (cm)** | **Tissue density (g/cm³)** | |
| --- | --- | --- | --- |
|  |  | **Mean** | **SD** |
| **Stem** | 2 - 4 | 0.19 | 0.03 |
|  | >4 - 5 | 0.20 | 0.02 |
|  | >5 - 6 | 0.21 | 0.04 |
|  | >6 - 7 | 0.19 | 0.03 |
|  | >7 - 8 | 0.22 | 0.04 |
|  | >8 | 0.27 | 0.05 |
| **Sheath** | 2 - 4 | 0.27 | 0.05 |
|  | >4 - 5 | 0.26 | 0.03 |
|  | >5 - 6 | 0.26 | 0.04 |
|  | >6 - 7 | 0.27 | 0.04 |
|  | >7 - 8 | 0.25 | 0.04 |
|  | >8 | 0.25 | 0.04 |
| **Petiole** | 2 - 4 | 0.22 | 0.03 |
|  | >4 - 5 | 0.21 | 0.02 |
|  | >5 - 6 | 0.21 | 0.03 |
|  | >6 - 7 | 0.21 | 0.02 |
|  | >7 - 8 | 0.20 | 0.03 |
|  | >8 | 0.20 | 0.02 |
| **Rachis** | >2 - 4 | 0.27 | 0.04 |
|  | >4 - 5 | 0.27 | 0.02 |
|  | >5 - 6 | 0.29 | 0.02 |
|  | >6 - 7 | 0.29 | 0.04 |
|  | >7 - 8 | 0.28 | 0.04 |
|  | >8 | 0.29 | 0.04 |
